# Protection of Urban Art Painting: A Laboratory Study

**DOI:** 10.3390/polym14010162

**Published:** 2021-12-31

**Authors:** Andrea Macchia, Sara Capriotti, Laura Rivaroli, Silvestro Antonio Ruffolo, Mauro Francesco La Russa

**Affiliations:** 1 Youth in Conservation of Cultural Heritage (YOCOCU), Via T. Tasso n.108, 00185 Rome, Italy; info@yococu.com (A.M.); saracapriotti@yahoo.com (S.C.); laura_rivaroli@yahoo.it (L.R.); 2 Department of Biology, Ecology and Earth Science, University of Calabria, Via Pietro Bucci, 87036 Arcavacata di Rende, Italy; mauro.larussa@unical.it

**Keywords:** anti-graffiti, protective layers, urban art, quartz paints, murals

## Abstract

Urban art is a form of artistic visual expression and communication that is created in the street and generally in the public dimension of urban spaces. Often these kinds of artworks are in outdoor environments, and they usually suffer from atmospheric weathering and anthropic vandalism. Recently, several strategies have been used to limit or remove the effects of such vandalism. Currently, the use of quartz paints is growing among artists; such paints after setting are more porous and rough on the surface with respect to regular paints. The aim of the study is to assess the performance of anti-graffiti coatings on quartz artworks paints. Two anti-graffiti products were chosen, and their behaviors were assessed in the laboratory by means of contact angle measurement, water capillary test, colorimetric analysis, and optical and electron microscopy. Results showed good water repellence efficacy of the tested products, demonstrating that they are suitable for the protection of urban art, but at least two applications on the surface are needed to achieve good performance.

## 1. Introduction

Urban art was born in XII century in a controversial environment and with a provocative spirit [1]. The choice of wall, outdoor, and large art is itself a choice of a public nature, in which the art is designed for show to everyone. These types of contemporary mural paintings are linked to the so-called “Public Art” thanks to the specific choice of artists, often representing politicized, ethical, and social messages directly in the street context [2]. In recent years, there has been a significant change in their recognition as a kind of public art that is commonly commissioned for the requalification of urban spaces and that must be preserved over time, which has given rise to scientific research and experiments to determine the best strategies for their conservation and restoration [3,4,5]. One of the most difficult problems for the conservation of urban art murals is their protection from vandalism actions that can affect and cover both small portions or the complete surface of the murals through *“crossatura”*, or simply vandalization [6]. Anti-graffiti coatings could be a solution to optimize urban art protection, because these treatments hinder the adhesion of the vandalism and its interaction with the underlying surface [7]. These coatings could cover the surface or penetrate through the pore system of the substrate, forming a protective barrier against the colorants and dyes contained in the sprays, markers, and other materials used to make graffiti [8,9,10].

The range of materials adopted in contemporary art is expanding, and the main chemical classes include alkyds, acrylics, styrene resins, and combinations thereof [11]. The literature reports several research projects aimed to identify appropriate anti-graffiti products used for the protection of cultural heritage [12], while fewer works are available on the use of anti-graffiti on contemporary murals [3,13].

In recent years, street artists have begun to use quartz paints, which are based on acrylic or siloxane polymers, and loaded with silica based particles, which provide a greater resistance to atmospheric agents, abrasion, and dirt. Moreover, such paints are more porous, and then their behavior in terms of wettability is quite different with respect to paints without inorganic fillers [13]. In this study, the performance of anti-graffiti coatings on a quartz paint was assessed.

Based on our previous research [3,13], two anti-graffiti products were chosen: PRO-ART, and AG09W. The first one is based on a fluorinated acrylic polymer, while AG09W is a mix of microcrystalline wax and of fluorinated polymers. Fluorinated polymers are able to provide good water and oil repellent properties to the treated surfaces, as well as good stability over time; this makes these materials very useful as protective coatings [14]. These anti-graffiti were applied in different amounts on specimens having a quartz paint on the top, and then laboratory tests were performed to assess the behavior toward water, aesthetical and colorimetric variations, and their ability to prevent or limit vandalism.

## 2. Materials and Methods

**Figure 1:**
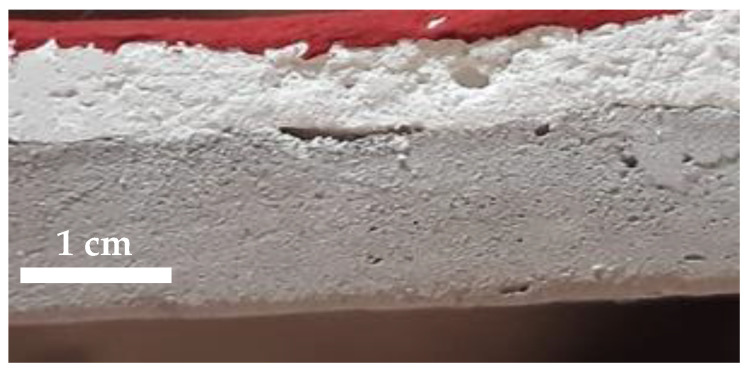
Stratigraphy of the specimens; from the bottom: concrete, plaster, painted layer.

To test the anti-graffiti products, 21 specimens were made to reproduce a substrate similar to those used by artists (Figure 1). In the first step, concrete cylinders (diameter 10 cm, height 2 cm) were made and left to dry for one month. Then, a plaster layer of about 1 cm was applied. After one month, the samples were painted with the quartz paint Ivas Superquarz Plus (Ivas Industria Vernici SpA, Forlì Cesena, Italy); it is based on an acryl-siloxane polymer and loaded with mineral particles having particle sizes lower than 100 microns.

Three samples were used as reference, while the others were coated by brush with two anti-graffiti coatings, PRO-ART (YOCOCU/Pelicoat, Rome, Italy) and AG09W (Keim, Diedorf, Germany) (Table 1), which are water based formulations and cannot solubilize the substrate. AG09W is commonly used for building heritage, while PRO-ART is specifically developed for urban art murals by YOCOCU (Youth in Conservation of Cultural Heritage). Both products were used in three different amounts on the specimens, namely one application, two applications and three applications (1 application = 200 mL/m^2^). Each application was repeated on three specimens.

Laboratory analyses were carried out to assess the performance of the anti-graffiti coatings. The evaluation of aesthetic variation caused by the application of the protective coatings was investigated qualitatively by means of a Dinolite AM411-FVW microscope (Dino-Lite Europe, Almere, The Netherlands), and the quantification of induced chromatic alteration was obtained by using a colorimeter (3hn Y3060, 3hn, Shenzhen, China). Colorimetric analyses were reported in the CIE L*a*b* space, where L* is the lightness/darkness coordinate, a* the red/green coordinate (+a* indicating red and −a* green), and b* the yellow/blue coordinate (+b* indicating yellow and −b* blue). The chromatic alterations were analyzed with the following equation:

(1) $$\Delta E=\sqrt{{\left(\Delta L^{*}\right)}^{2}+{\left(\Delta a^{*}\right)}^{2}+{\left(\Delta b^{*}\right)}^{2}}$$

where ΔL*, Δa*, and Δb* are the expressions for color differences between the color parameters of surface before and after protective layer application.

To investigate the distribution and of the coating on the specimen surfaces, ultraviolet fluorescence imaging was acquired using a Madatec Imaging System with an NX500 28.2 MP BSI CMOS camera and 365 nm UV light, and a yellow -495- 52 mm F-PRO MRC 022 filter.

The ability of PRO-ART and of AG09W to protect the surface was analyzed by means of contact angle and capillary absorption measurements. 

The contact angle measurements were carried out as follows [15]: about 10 µL of deionized water drops were laid down on five different spots. Then, drop shapes were recorded with a camera; the tangent (angle) of the drop with the stone surface was measured.

In order to evaluate the amount of water absorbed by specimen per surface unit over time (A), measurements of water absorption were performed by capillarity test [16]:

A = (P_i_ − P_0_)/S (2)

where S is the area of the base of the sample in contact with water, and P_i_ and P_0_ are the sample weights measured during the test, respectively, at the time t and the time 0.

After performing the abovementioned non-destructive tests, specimens treated with 3 application of anti-graffiti coating were dried at 60 °C for 24 h, and then a black paint (Montana 94 Black, Barcelona, Spain) was applied to simulate a vandal action on an urban art painting. After drying, the specimens were cut and observed in cross section by means of a scanning electron microscope (SEM) (Tescan Scanning Electron Microscope, Voltage 20 kV, beam current: 0.2 mA, Brno, Czech Republic).

## 3. Results and Discussion

**Figure 2:**
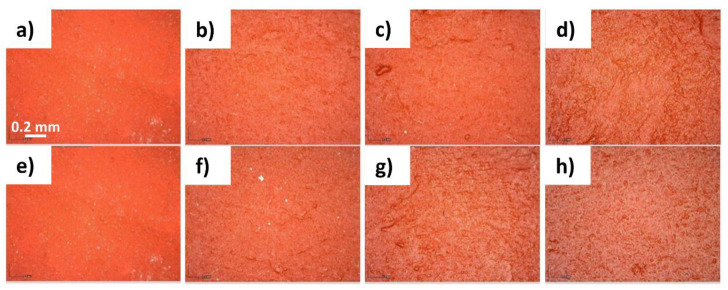
Images of untreated red painted surfaces (**a**,**e**); surfaces treated with PRO-ART with (**b**) 1 application, (**c**) 2 applications, and (**d**) 3 applications; and surfaces treated with AG09W with (**f**) 1 application, (**g**) 2 applications, and (**h**) 3 applications.

The evaluation of aesthetic variation caused by the application of protective coatings was investigated by the analysis with Dinolite (Figure 2). PRO-ART determined a slight variation of the surface after the third application, causing a slight darkening. AG09W induced a darkening of the red paint after the second application. Both PRO-ART and especially AG09W created a glossy surface.

As seen in Table 2, the values of ∆E increased as the number of applications increased, especially for AG09W; however, it is worth noting that the ∆E value was always below 5, the threshold generally considered acceptable in the field of conservation of cultural heritage.

**Figure 3:**
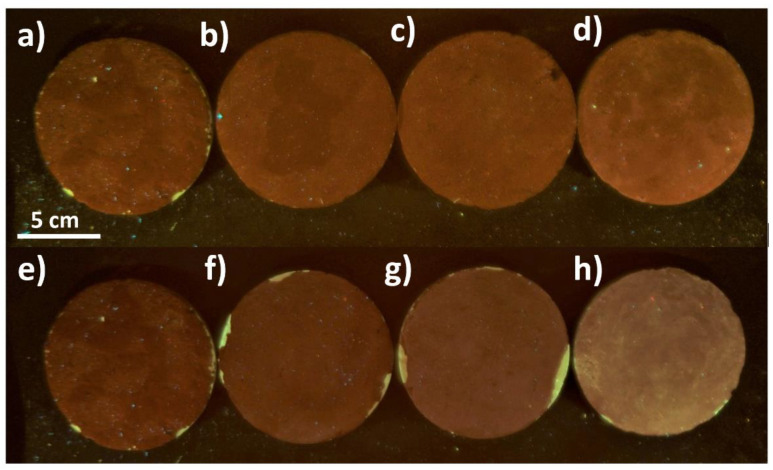
Photographs of samples under UV light: untreated (**a**,**e**); treated with PRO-ART with (**b**) 1 application, (**c**) 2 applications, and (**d**) 3 applications; and treated with AG09W with (**f**) 1 application, (**g**) 2 applications, and (**h**) 3 applications.

Moreover, PRO-ART seemed to induce a lower chromatic alteration with a lower chromatic difference between the numbers of applications of the product. Multispectral analyses were performed to study UV fluorescence and to verify some differences between PRO-ART and AG09W products, as well as the differences due to the number of protective coating applications (Figure 3). Both PRO-ART and AG09W formed a homogenous coating on the surface. For PRO-ART (Figure 3a–d) there was a gradual increase in the fluorescent component, especially visible in the sample with three applications of the protective coating. Samples coated with AG09W (Figure 3e–h) revealed a strong fluorescent component, particularly detectable on the three-times-treated surface.

**Figure 4:**
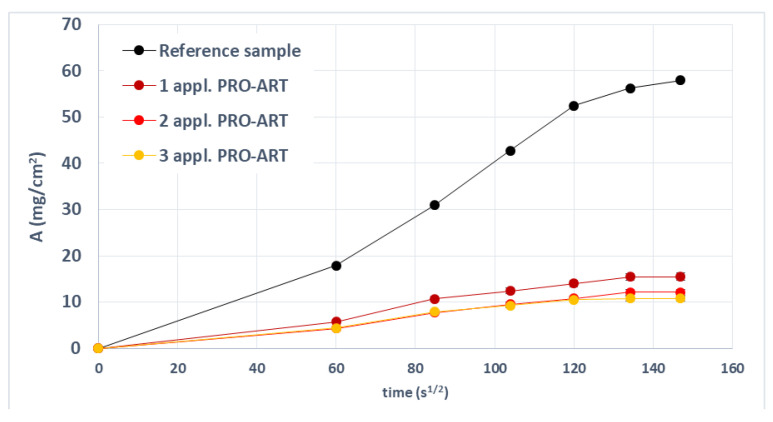
Absorbance over time detected on samples uncoated and coated with PRO-ART.

**Figure 5:**
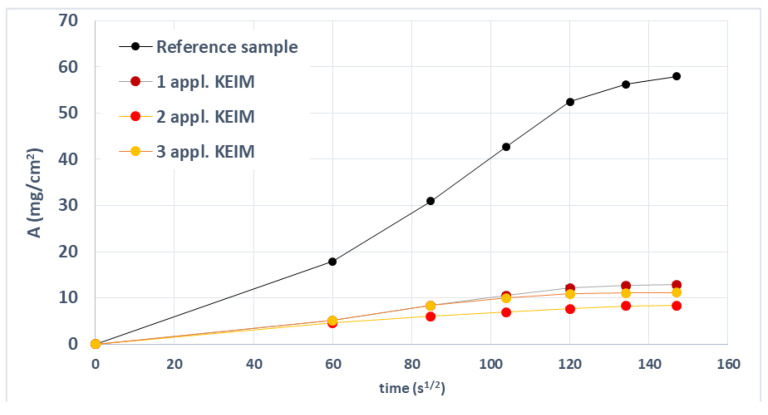
Absorbance over time detected on samples uncoated and coated with AG09W.

The effectiveness of PRO-ART and AG09W in repelling water absorbed by capillary action was evaluated by comparing treated and untreated samples. Figure 4 shows the results of PRO-ART treatment. The product led to a certain level of water repellency from the first application. All treatments samples showed a very similar trend. The specimens treated with AG09W showed a behavior very similar to the PRO-ART treatment (Figure 5).

**Figure 6:**
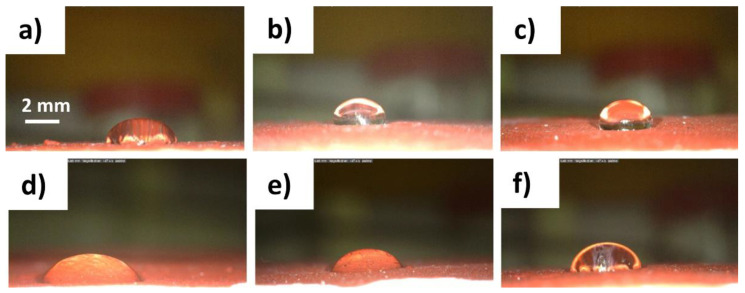
Images of contact angle tests: (**a**) 1 application, (**b**) 2 applications, and (**c**) 3 applications of PRO-ART; and (**d**) 1 application, (**e**) 2 applications, and (**f**) 3 applications of AG09W.

The hydrophobicity of the treated surfaces was checked by contact angle measurements (Figure 6 and Table 3). The values of contact angle increased as the amount of anti-graffiti increased on the treated surfaces. After three applications, PRO-ART induced a contact angle of about 105°, while in the case of AG09W, a lower value was reached (83°), which suggests a lower wettability of the PRO-ART coated surface.

**Figure 7:**
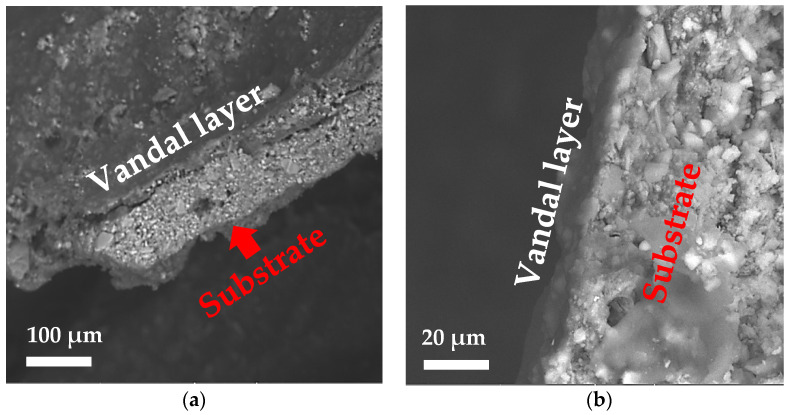
SEM images of samples coated with 3 applications of (**a**)PRO-ART and (**b**) AG09W and vandalized with black acrylic paint.

SEM images allowed the interaction between coatings and vandal paint to be assessed. In Figure 7 is shown the stratigraphy of specimens treated 3 times with PRO-ART and AG09W and vandalized with black spray paint. In the case of PRO-ART (Figure 7a), the vandal layer appeared not adherent on the underlying surface, suggesting a good action of the anti-graffiti in preventing the adhesion of the vandal layer. On the contrary, the surface coated with AG09W seemed to suffer from the adhesion of the vandal paint, since there was not any visible discontinuity; it seemed to be rather a penetration of the vandal layer in the substrate.

## 4. Conclusions

The conservation of urban art from vandalism still represents a challenging task. The use of anti-graffiti products can represent a powerful tool for preventive conservation of street art, since those materials made it possible to remove a vandalism layer. In this research the behavior of two anti-graffiti products made with quartz paints was tested, namely PRO-ART (based on a fluorinated acrylic polymer) and AG09W (a mix of microcrystalline wax and fluorinated polymers), for the protection of artworks against vandalism. Specimens were made in the laboratory, and both products were applied in three different amounts. Results showed good water repellence efficacy of the two products, demonstrating that both of them are suitable for the protection of urban art; at least two applications on the surface are needed to achieve a good performance. However, colorimetric assessments showed better behavior of PRO-ART, since it induced lower color alteration. Moreover, on a microscopic scale, on surfaces treated with PRO-ART, scarce adhesion of the vandal paint was observed, which suggests good efficiency of the anti-graffiti feature. This research suggests that the use of anti-graffiti coatings can provide a protection feature to street art artwork made of quartz paint; however, further studies will be aimed to assess the durability of those protective coatings as well as to develop experimentations on “real cases”.

## Figures and Tables

**Table 1:** Anti-graffiti coatings studied in the experimentation.

Composition	Product Name	Producer	Type
Fluorinated acrylic	PRO-ART	YOCOCU/Pelicoat	Permanent
Mix of microcrystalline wax and of fluorinated polymers	AG09W	Keim	Sacrificial

**Table 2:** Chromatic alteration induced by the coatings on surfaces.

	∆E (Coated—Untreated Surface)		
	1 Appl.	2 Appl.	3 Appl.
PRO-ART	1.5 ± 0.3	2.2 ± 0.6	2.5 ± 1.0
AG09W	1.6 ± 0.7	3.1 ± 0.5	4.1 ± 1.2

**Table 3:** Contact angle measurements.

ID	Contact Angle (°)		
	1 Appl.	2 Appl.	3 Appl.
PRO-ART	74.5 ± 5.0	96.9 ± 5.4	105.0 ± 4.6
AG09W	64.3 ± 7.1	72.1 ± 6.3	83.3 ± 4.2

## Data Availability

Not applicable.
